# Transferring Key Success Factors from Ambulatory Care into the Community Pharmacy in the United States

**DOI:** 10.3390/pharmacy9030116

**Published:** 2021-06-23

**Authors:** Alex J. Luli, Linda Awdishu, Jan D. Hirsch, Jonathan H. Watanabe, Mark Bounthavong, Candis M. Morello

**Affiliations:** 1 Skaggs School of Pharmacy and Pharmaceutical Sciences, University of California San Diego, 9500 Gilman Drive, La Jolla, CA 92093, USA; lawdishu@health.ucsd.edu (L.A.); mbounthavong@health.ucsd.edu (M.B.); cmmorello@health.ucsd.edu (C.M.M.); 2 Department of Clinical Pharmacy Practice, School of Pharmacy and Pharmaceutical Sciences, University of California Irvine, 101 Theory, Suite 100, Irvine, CA 92612, USA; jdhirsch@uci.edu (J.D.H.); jonathan.watanabe@uci.edu (J.H.W.)

**Keywords:** pharmacist, patient care services, community-based pharmacy practice, ambulatory care

## Abstract

In the United States, pharmacists’ scope of practice continues to expand, with increasing opportunities for pharmacists in all practice settings to enhance health in society. In ambulatory care, pharmacists remain integral members on the healthcare team and have demonstrated positive impacts on patient care. Sharing similar characteristics as pharmacists in the community setting, a deeper look into common elements of a successful ambulatory care practice that can be applied in the community pharmacy setting is warranted. Key success factors identified from ambulatory care include (1) maximizing a pharmacist’s unique knowledge base and skill set, (2) forming collaborations with physicians and other providers, (3) demonstrating outcomes and value, and (4) maintaining sustainability. Opportunities exist for pharmacists in the community setting to utilize these success factors when developing, implementing, and/or expanding direct patient care services that improve accessibility to quality care and population health.

## 1. Introduction

In the United State, the scope of pharmacists’ practice continues to expand, including opportunities for pharmacists to play an increasingly important role in patient care and public health. This shift has been propelled recently due to the urgency of the severe acute respiratory syndrome coronavirus 2 (SARS-CoV-2) pandemic that began in early 2020 [1]. Across the world, pharmacists took on expanded key roles to reduce the impact of the virus—including administering vaccines, preparation of hand and surface disinfectants, renewing chronic treatment prescriptions, and performing coronavirus disease 2019 (COVID-19) tests [2]. Recognizing the importance of community pharmacies in delivering vaccines, the United States Department of Health and Human Services announced a partnership with pharmacy chains designed to maximize access to COVID-19 vaccinations once available [3]. Even before the SARS-CoV-2 pandemic, pharmacists have been recognized for their role in advancing important public health measures [4]. For example, pharmacy-based immunization services are now widely available in many community pharmacies and offer patients a convenient outlet to receive routine vaccines [5,6]. Pharmacists have also demonstrated positive impacts in the areas of tobacco cessation, administering hormonal contraception, and the management of chronic diseases such as hypertension, diabetes, and chronic obstructive pulmonary disease (COPD) [7,8,9,10,11]. Pharmacists continue to be one of the most accessible healthcare providers for Americans. More than 8 in 10 Americans live within 5 miles of a pharmacy, and many visit their community pharmacy more than their physician office [12]. This accessibility, along with a recognized role supporting public health measures, patient care, and improving outcomes, has helped drive new opportunities for community pharmacists.

Despite these opportunities, challenges remain in expanding patient care services in the community pharmacy setting [13]. Although the expanded scope of professional practice can be seen as an important step towards development of new services, advances are needed in how pharmacists are compensated for delivering care. For example, Centers for Medicare and Medicaid Services (CMS) have yet to recognize pharmacists as providers, and therefore pharmacists remain unable to bill CMS for healthcare services provided to beneficiaries [14]. Pharmacies today must rely mostly on revenue from dispensing prescription medications, even though reimbursement for this has remained low [15]. Further, no standard for documentation of pharmacist services currently exists. CMS, for example, has documentation requirements that must be completed when providing medication therapy management (MTM) for beneficiaries (i.e., completion of a medication action plan), but for many pharmacy services these requirements do not exist. In addition, although pharmacy software may have capabilities to document services internally, they may not be able to communicate this to external organizations (e.g., a progress note created in a pharmacy will not be shared with an outside physician group).

Although some of these challenges will take major shifts in policy, regulation, and infrastructure to overcome (such as physical space to deliver care, number of support staff, and regulation of pharmacy benefit managers), many others can be addressed. Recently, the 2019 National Pharmacy Workforce Study indicated that many practicing pharmacists provide limited direct patient care outside of dispensing activities. Pharmacists across settings reported spending an average time of 49% on dispensing activities and 22% on non-dispensing activities [16]. The most common non-dispensing services provided by practicing pharmacists differed depending on practice setting (Table 1). Approximately two-thirds of community pharmacists report MTM as a commonly provided service. Given the similarities in common services provided in both the community and ambulatory care settings (i.e., MTM, education, and counseling), key lessons from ambulatory care practice development can be applied to the community setting in an effort to expand patient care activities.

In ambulatory care settings, pharmacists have established themselves as integral members of the healthcare team [17]. Even though each practice setting has unique characteristics that are important to recognize, understand, and utilize, many qualities of a successful pharmacist practice can be identified and applied broadly. For example, at the Veterans Affairs San Diego Healthcare System (VASDHS), a collaboration between a pharmacist and endocrinologist in a diabetes intense medical management (DIMM) clinic for complex patients with type 2 diabetes (T2D) resulted in significantly improved glycosylated hemoglobin A1c measurements compared to usual care [18]. Many factors contributed to the success of this program, such as utilizing a pharmacist’s unique skill set in medication management, identifying and collaborating with a physician champion (endocrinologist), and measuring key clinical outcomes (e.g., hemoglobin A1c).

Despite similar challenges of limited reimbursement options, pharmacists practicing in ambulatory care settings continue to grow. Citing the need to shift resources to help lower costs and improve patient care, the American Society of Health-System Pharmacists (ASHP) Pharmacy Forecast 2016–2020 report found that 69% of forecast panelists predicted that at least 25% of health systems will transition 10% or more of inpatient positions to ambulatory care [19]. Further, in the ASHP annual Pharmacy Forecast 2020 report, 82% of pharmacy leaders indicated their organizations offered ambulatory care services [20]. In this paper, we aimed to describe and elaborate on key factors that have led to successful implementation and expansion of pharmacist services in ambulatory care settings, and how these factors can be translated and applied in community pharmacies in the U.S. Although the following commentary is based on experiences and examples operating in the unique U.S. healthcare system, each “success factor” can be broadly applied to different healthcare systems across the world. We encourage a similar thought exercise for those operating in a different system and hope this may serve as a blueprint for that endeavor.

## 2. Success Factor 1: Specialized Medication Knowledge and Skills

Pharmacists have a knowledge base and skill set that is unique among healthcare providers. Today, pharmacists are licensed professionals who have completed a doctoral degree, accredited postgraduate clinical training, or the equivalent clinical experience and are practicing in diverse settings, including team-based, direct patient care environments [21,22]. Many pharmacists further enhance their unique knowledge base by obtaining board certification with the Board of Pharmacy Specialties (BPS). Currently, there are fourteen distinct clinical specialties, including ambulatory care [23]. Moreover, numerous U.S. states have enacted legislation to create advanced practice pharmacist designations that provide unique authorities, such as performing patient assessments and ordering drug-therapy related labs [24]. The American College of Clinical Pharmacy published a set of core competencies that defines the required knowledge and skills of pharmacists and serves as a basis for assessment of competence. The six core competencies include: (1) direct patient care, (2) pharmacotherapy knowledge, (3) systems-based care and population health, (4) communication, (5) professionalism, and (6) continuing professional development [25]. These competencies are analogous to the Accreditation Council for Graduate Medical Education core competencies of (1) patient care and procedural skills, (2) medical knowledge, (3) systems-based practice, (4) interpersonal and communication skills, (5) professionalism, and (6) practice-based learning and improvement [26]. While mostly similar to physician competencies, pharmacist competencies also focus on achieving optimal medication therapy outcomes. Further, pharmacists undergo extensive communication training, including motivational interviewing and cultural competency, to enhance their effectiveness as a patient educator. These are the characteristics that best position the pharmacist to assess medication efficacy, safety, adherence, affordability, and provide overall optimization of a patient’s medication regimen. In the DIMM clinic example cited above, a pharmacist-certified diabetes educator (CDE) was responsible for patient MTM and real-time diabetes education during a 60 min clinic visit. Matching the needs of the patient population (e.g., patients with T2D with an A1C > 9%) to an experienced, skilled, and knowledgeable pharmacist set the foundation for positive patient outcomes [18].

There are still several factors that may impact the integration of pharmacists into ambulatory care practices, including referrals to pharmacists, scheduling and logistics, access to electronic health records, in-person communication with the physician, and lack of payment for services [27]. Access to the electronic health record, in-person communication, and limited reimbursement are also important barriers faced by the community pharmacist. However, ambulatory care pharmacists have overcome these barriers and utilized their medication knowledge and skills to develop clinical services with positive patient outcomes [28,29,30]. Lessons learned from ambulatory care pharmacist experience and outcomes can be applied to expand services within community pharmacies.

Pharmacists have identified ways to utilize their unique skills in the community pharmacy setting, particularly around providing medication therapy management (MTM). MTM services have been offered in many community pharmacies in the US, aided by requirements from the Centers for Medicare and Medicaid Services (CMS) that mandate that Part D plan sponsors must incorporate MTM programs into their benefit structures [31]. MTM services make use of the pharmacist’s skill set in assessing the safety and efficacy of a patient’s medication regimen, while leveraging pharmacist accessibility to community-dwelling patients. Many examples exist that indicate the positive impact MTM services can have on economic and clinically important outcomes [32,33,34]. Other examples of utilizing a pharmacist’s unique skills and accessibility include pharmacist-administered medications [35], tobacco cessation programs [36], wellness coaching [37], and travel health services [38,39]. The future will likely include other areas in which pharmacists can utilize their specialized skills. For example, as more is understood about how genes can affect drug response and therapeutic outcomes (i.e., pharmacogenomics), and point-of-care testing becomes cheaper and widespread, pharmacists are well positioned to establish pharmacogenomic services [40]. Thus, the unique skill set of the pharmacist should remain an important consideration as pharmacists look to expand services within the community setting.

## 3. Success Factor 2: Collaborations and Clinical Practice Agreements

Collaboration is an essential success factor in many areas of healthcare. Since the patient care continuum can involve many different providers in various settings, it is important for providers to work together. Team-based care has been shown to optimize workflow, enhance quality, and improve patient outcomes [41]. From a business perspective, aligning vision, goals, and incentives with different stakeholders across settings is important to develop and sustain patient care services. For pharmacists in any setting, understanding factors that create or enhance collaboration is vital. One common example of this in practice is establishing a formal collaborative practice agreement (CPA) that outlines roles and responsibilities of each party involved. The American Academy of Family Physicians supports collaborations between pharmacists and physicians to improve patient care [42]. Further, the Centers for Disease Control and Prevention (CDC), with support from American Pharmacists Association, American Medical Association, and American Association of Nurse Practitioners, has created a resource to help develop and execute these collaborations [43]. Even for pharmacists operating with established prescribing authorities, collaborations are a key factor for starting, operating, and maintaining robust patient care services.

Engaging pharmacists in collaborative patient care, regardless of the setting, is a multistep process. First, a needs assessment should be conducted for each practice site to identify potential gaps in patient care and determine matching skill sets of pharmacist providers. In ambulatory care settings, starting points may include services for patients with common chronic diseases such as asthma, cardiovascular disease, hypertension, and diabetes. These are typically high need areas where patient volume and increased complexity exceed existing provider capacities [44]. In addition, transitions of care from hospital discharge to home, or skilled nursing facility to home, also represent high risk periods for errors and require high resource utilization. Pharmacist services in ambulatory settings have been able to help fill these gaps. Community settings also have great potential to improve transitions of care and may even be better suited to provide these services given the accessibility and geographic proximity of pharmacies to patient homes. For example, there have been positive reports that MTM services provided directly after hospital discharge can help reduce readmission rates [45,46].

Once this needs assessment is complete, identifying a physician champion is a key factor to endorse, support, and promote new pharmacist services [47]. Depending on the area of need or patient population, a physician practicing in that area is a natural starting point. For example, when developing an interprofessional clinic for chronic kidney disease patients, identifying a nephrologist would be appropriate. Physician champions may also be identified in other ways, such as through daily clinical interactions. This relationship can be fostered by having an in-person meeting to discuss common goals. Some pharmacists have found it useful to spend time in the physician’s practice setting so the pair can identify optimal communication styles and practice approaches. In addition to helping the pharmacist, this can also benefit the physician’s practice by improving clinical outcomes, simplifying workflow, and improving patient satisfaction [48]. Trustworthiness, relationship initiation, and role specification are important to establishing an effective collaborative working relationship with physicians [49].

In community pharmacy, a similar approach can be adopted. Although there is less potential for face-to-face interaction compared to ambulatory care settings, a thorough needs assessment and the specific service being implemented can serve as a path to identifying an appropriate physician champion. For example, when implementing a CDC-accredited diabetes prevention program (DPP), a community pharmacy in Pennsylvania utilized partners with similar interests to enhance their program, including a physician group [50]. Many services that provide preventive care measures, such as a DPP, would likely find a physician champion from nearby primary care practices, due to their overlapping missions on keeping patients healthy [51].

Once identified, the pharmacist can develop a CPA with the physician champion. While many pharmacist services are already covered within a pharmacists’ scope of practice, initiating a CPA delineates clear roles, clinic or treatment goals, and measurable outcomes, as well as establishing a degree of trust and open communication [43]. The CPA is a formal agreement between the physician provider, who makes a clinical diagnosis, and the pharmacist. It also defines the scope of practice and enables the pharmacist to deliver medication management, acting as a physician extender. Since state requirements vary, the terms and functions within the CPA should comply with state law. The main sections of a CPA usually include the agreed upon scope of patient care, (i.e., disease management protocols based on national guidelines, patient inclusion criteria), legal issues (i.e., liability insurance, CPA duration), and administrative components (i.e., clinical care space, budget, documentation, record keeping, training/education, quality assurance and outcome measures). Several CPA templates are available, with some specifically designed for community pharmacy practice settings [43,52].

In summary, effective collaborations are key for the creation, development, marketing, and expansion of comprehensive services, in addition to dissemination of important outcomes.

## 4. Success Factor 3: Demonstrating Outcomes and Value

Pharmacists must demonstrate how their patient care services produce outcomes that are of value to all stakeholders. Value can be defined in multiple ways and depends largely on the context in which it is defined. In general, an intervention is valuable if the outcomes produced are of greater perceived value than the resources used to produce it. There are many types of outcomes that may be considered valuable. A useful model for considering the value of pharmacist services is the economic, clinical, and humanistic outcomes (ECHO) model [53,54]. Economic outcomes could be any direct costs saved due to the intervention (e.g., lower medication costs, fewer physician office visits or hospitalizations) and/or indirect costs framed as productivity gains (e.g., fewer workdays lost due to illness, greater productivity at work due to improved symptoms). The monetary value of these economic outcomes can be compared to the cost of the intervention to determine the cost-effectiveness of the service and return on investment (ROI). Clinical outcomes include those that occur as a result of the disease or treatment and can be affected by the intervention—such as reporting percent of patients with T2D that meet hemoglobin A1c, blood pressure, and lipid goals. Returning to the DIMM clinic example, clinical outcomes included not only A1c, but also blood pressure, lipids, and weight, since these are also important clinical markers in T2D patient populations. Finally, humanistic outcomes consider the effects of an intervention on a patient’s satisfaction, quality of life, or preferences. This may include the patient’s rating of their general health or well-being, and/or patient satisfaction with the service or the pharmacist provider.

Determining the types of outcomes to collect, assess, and report for a new patient care service can be confusing. A useful tool for considering the key elements needed to demonstrate value is the Pharmacist Patient Care Intervention Reporting (PaCIR) checklist. The PaCIR is a convenient checklist designed for reporting results of pharmacist patient care service interventions [55]. It can also be used to guide the design of interventions to keep the end point of reporting value results clearly in mind for all stakeholders. While all nine elements of the checklist are useful for reporting, three are particularly useful for designing interventions that will clearly demonstrate value and outcomes. These include (1) carefully selecting and defining the patient population, (2) being intentional in selection of data to collect, and (3) considering the intensity and cost of the intervention vs. expected value of outcomes delivered. With regard to the patient population, it is important to identify those with high value to the organization that can be matched to available pharmacist resources within the pharmacy, and outcomes that are amenable to pharmacist interventions with a high likelihood of producing positive outcomes in a short period of time. Of note, while more severe or complex patients may appear to be a high-value group, a pharmacist service intervention alone may not impact outcomes being controlled primarily by other factors. Once a patient population and the corresponding pharmacist service are selected, the intentional selection of outcomes to assess should include those that will demonstrate value to all stakeholders (e.g., clinical for clinicians, savings for finance, adverse event prevention for risk management). Clearly stating and agreeing on the proposed value proposition amongst all stakeholders up front is a key element for designing a successful intervention. Clear outcomes with defined measures and targets are essential since some outcomes may require increased utilization or resources (e.g., medication utilization may increase in order to achieve improved clinical outcomes, and lower overall cost of patient’s care). Choosing easily obtainable metrics that are reliable and valid, and preferably part of normal data collection and documentation processes of the organization, will help simplify data collection and align with organizational priorities. Finally, the intensity and cost of the intervention should be carefully planned to be less than, or proportionate to, the expected value of outcomes delivered. A key is to use all resources in their most cost-effective manner. Salary and benefits of pharmacists and staff are likely the costliest elements of any pharmacy services intervention, emphasizing the deliberate and judicious use of staff as the intervention cost is a key driver of cost effectiveness and ROI for the service. Therefore, it is imperative to define the clinical care processes involved in the intervention and position pharmacists to fill roles that utilize their unique medication knowledge and skills, while seeking out opportunities for less costly members of the team to perform non-clinical or less complex tasks. Matching the service cadence (i.e., frequency and duration) with the natural disease progression, expected effects, and patient needs, is also an important aspect of designing an intervention. For example, an intervention in diabetes management that lasts one month with A1c as an outcome measure, will not demonstrate the full impact of the intervention since A1c is unlikely to change within a one-month period.

Similarly, community pharmacies should consider the value and outcomes new or existing services will create. As mentioned, incorporating these principles early in the service development process can help ensure meaningful outcomes. Considerations include the number of outcomes feasible to measure and which outcomes are most important and/or relevant. For example, if a service is targeted to high-risk patients with multiple medications and comorbidities, the outcomes measured should consider the amount of effort needed from the pharmacist (e.g., longer duration of visits, more frequent interactions), since that will affect overall costs and when results can be demonstrated. In order to maximize cost-effectiveness, pharmacists in the community setting can look to their support staff. Pharmacy technicians can be a key resource to ensuring cost-effectiveness of any service and expanded roles for technicians are emerging. In the U.S., some states now allow pharmacy technicians to perform prescription verification [56], administer immunizations [57], and provide medication reconciliation [58]. Utilizing technicians for technical tasks will then allow the pharmacist to spend time on activities that require clinical judgement. Barriers remain to utilize technicians however, including inconsistency in training requirements and education [59], as well as low willingness among technicians to perform these activities [60]. Moreover, pharmacy technician wages will need to match the increased responsibilities and burden. Since 2008, pharmacy technicians have experienced negative wage premiums [61], an indication that they are not properly compensated for their current duties. Additional opportunities to streamline workflow and increase efficiency in the community pharmacy should be considered, such as employing medication synchronization and an appointment-based model. Often used together, both tools can help pharmacists stay focused on clinically related tasks, and may help improve patient outcomes, such as medication adherence [62,63,64].

Demonstrating outcomes and value are key success factors when developing pharmacist-provided services, in both ambulatory and community settings. It is important to use established tools that can help to ensure needs are met by all stakeholders.

## 5. Success Factor 4: Sustainability

Finally, financial sustainability for pharmacist services is imperative in both ambulatory care and community settings. Many health systems have realized the benefits of including ambulatory care pharmacists in patient care roles. This adoption has expanded, with further emphasis on value-based care and improved patient outcomes as the impetus for inclusion of pharmacists in direct patient care [65,66,67]. The Collaboration Among Pharmacists and Physicians to Improve Outcomes Now (CAPTION) study, a prospective, cluster-randomized trial fueled by an interest in examining benefits of embedded clinical pharmacists in bolstering hypertension management demonstrated a large improvement in achievement of blood pressure goals at 9 months [68]. The authors concluded that as federal payers continue the migration toward payment tied to population-based outcomes, sustainable financial models for pharmacist practitioners will consist of a mix of population-based payments based on team-configured care for the plan, health system, or organization in which they are employed.

In the community pharmacy setting, direct-care services must be affirmed for scale and longevity of community pharmacy-based care. Prior research has demonstrated that patients are willing to pay additional out-of-pocket costs for the convenience, accessibility, and comfort of care in a community pharmacy. An analysis of 89,011 privately paying patients across 479 pharmacies in England found that many patients were willing pay for an immunization in a pharmacy even if they could receive it for free in a physician’s office [69]. Further, care providers at retail clinics, usually operated by large pharmacy chains, are capable of providing quality care for routine ailments at reduced cost compared to urgent care clinics, physician offices, and emergency departments [70]. While examples of pharmacist services reimbursed in the community setting do exist, the bulk are tied explicitly to furnishing of a product (vaccinations, diagnostic testing, lipid monitoring, etc.). There are efforts to change this. A novel 2017 pilot program in Washington State among twelve community chain pharmacies deployed a value-based payment model predicated on payments categorically tied to improvements scheduled to a tiered incentive structure [71]. The program demonstrated an improvement in influenza rates for all twelve value-based incentive pilot pharmacies. Management of the pharmacies received quarterly reports of their immunization performance and earnings from the payment models during this time, similar to what one would expect from an insurance-based program. Value-based incentive earnings were paid to each community pharmacy partner quarterly at the corporate level. Hence, billing operations were not needed in the pharmacy since payment occurred after evaluation of outcomes. Some community pharmacies have succeeded in arranging billable service arrangements for non-dispensing services [72]. However, these are not seen as a revenue driver necessary to sustain the overall operation.

It is unclear how a fee-based framework for pharmacist-delivered services can be self-sustaining within today’s pharmacy reimbursement system. Fortuitously, the US healthcare system is at a crossroad in terms of payment based on episodic care. Recent movement by large insurers to pivot away from fee-for-service structures towards bundled payment mechanisms that reward clinical services that improve outcomes and conserve dollars provides a significant opportunity for pharmacists [73]. The two largest pharmacy chains in the US are both integrated with insurers and operate embedded clinics in many locations. Pharmacist-directed care clinics make sense for provision of services that deliver high-value, longitudinal care that reduces expensive emergency department visits and hospitalizations that preserve the highest margins on capitated payments. This aligns achievement of clinical outcomes with financial stability for clinicians, payers, and purchasers/employers. Some individual community pharmacies in the US have recognized this with the development of clinically integrated pharmacy networks, committed to delivering high quality, evidence-based, patient-centered care to reduce costs and improve outcomes [74,75].

Further, many employers are developing their own clinics to lower health care spending while improving employee health and productivity. Pharmacists must continue pursuing ongoing efforts to coordinate with employer groups to establish community pharmacy-based clinics that can deliver care for workers. The recently released “Employer Toolkit” by the non-profit Get the Medications Right Institute provides guidance on constructing frameworks to bolster medication management services for employers through actionable approaches with pharmacy benefit managers (PBMs), medical carriers, benefit consultants, and employees [76]. Given the massive employer-based health insurance system, the US, more so than any developed nation, links employers to societal health. This framework must be leveraged to enhance patient care outcomes through clinical pharmacy services supported by employers.

Sustainability of any healthcare service represents a common challenge. Hospitals and health systems continue to sustain the utilization of ambulatory care pharmacists to control costs and improve care. With increased avenues for revenue outside of dispensing-related activities, community pharmacy is well positioned to take advantage of these opportunities.

## 6. Conclusions

Using the above key success factors in the community setting can help propel pharmacy practice forward. Although inherent differences exist between ambulatory and community practice settings, key factors from successful examples of developing, expanding, and sustaining pharmacist services can be applied across these unique settings. Community pharmacies have the potential to create sustainable revenue to support patient care services by leveraging their unique position and accessibility to the patient. Further, patients will benefit from additional locations to receive convenient, quality care. Future directions include further involvement with value-based incentives, promotion and collaboration with public health initiatives, and establishing the community pharmacy as a multi-faceted, healthcare service hub.

## Figures and Tables

**Table 1:** Most common services offered by practice setting, 2019 [16].

Practice Setting	Service Offered	% of Pharmacists Offering
Hospital/Acute Care	Drug level monitoring	87.2%
	Therapeutic drug interchange	81.5%
	Ordering lab tests	72.7%
Ambulatory Care	Medication education	61.6%
	Counseling	48.5%
	Changing drug therapy independently	45.1%
Community	Vaccinations	90.0%
	Medication assistance	83.4%
	Medication therapy management	66.7%

## Data Availability

Not applicable.
